# Late-onset major depressive disorder: exploring the therapeutic potential of enhancing cerebral brain-derived neurotrophic factor expression through targeted microRNA delivery

**DOI:** 10.1038/s41398-024-02935-7

**Published:** 2024-09-03

**Authors:** Giovanni Lai, Marco Malavolta, Serena Marcozzi, Giorgia Bigossi, Maria Elisa Giuliani, Tiziana Casoli, Marta Balietti

**Affiliations:** 1 Advanced Technology Center for Aging Research and Geriatric Mouse Clinic, IRCCS INRCA, Ancona, Italy; 2 Center of Neurobiology of Aging, IRCCS INRCA, Ancona, Italy

**Keywords:** Depression, Epigenetics and behaviour

## Abstract

Major depressive disorder (MDD) is a severe psychiatric condition that significantly impacts the overall quality of life. Although MDD can occur across all age groups, it is notably prevalent among older individuals, with the aggravating circumstance that the clinical condition is frequently overlooked and undertreated. Furthermore, older adults often encounter resistance to standard treatments, experience adverse events, and face challenges associated with polypharmacy. Given that late-life MDD is associated with heightened rates of disability and mortality, as well as imposing a significant economic and logistical burden on healthcare systems, it becomes imperative to explore novel therapeutic approaches. These could serve as either supplements to standard guidelines or alternatives for non-responsive patients, potentially enhancing the management of geriatric MDD patients. This review aims to delve into the potential of microRNAs targeting Brain-Derived Neurotrophic Factor (BDNF). In MDD, a significant decrease in both central and peripheral BDNF has been well-documented, raising implications for therapy response. Notably, BDNF appears to be a key player in the intricate interplay between microRNA-induced neuroplasticity deficits and neuroinflammation, both processes deeply implicated in the onset and progression of the disease. Special emphasis is placed on delivery methods, with a comprehensive comparison of the strengths and weaknesses of each proposed approach. Our hypothesis proposes that employing multiple microRNAs concurrently, with the ability to directly influence BDNF and activate closely associated pathways, may represent the most promising strategy. Regarding vehicles, although the perfect nanoparticle remains elusive, considering the trade-offs, liposomes emerge as the most suitable option.

## Introduction

Major depressive disorder (MDD) is a severe psychiatric condition characterized by symptoms such as anhedonia, feelings of worthlessness, impaired cognitive function, and disrupted sleep patterns, all of which significantly affect the overall quality of life [1, 2]. Although depression can occur across all age groups, it is notably prevalent among older individuals, with the aggravating circumstance that the clinical condition is frequently overlooked and undertreated [3]. According to recent meta-analyses, the worldwide prevalence of depression in older adults varies from 28.4% to 35.1%, with differences linked to geographical regions and diagnostic tools [4, 5].

In older adults, unlike in other age groups, depressive symptoms are often part of a multimorbidity condition. This significantly increases the risk of mortality [6], can complicate pharmacological management [7], may lead clinicians to underestimate the importance of treating depression while focusing on other chronic pathologies [8], and can exacerbate the reduction of independent life [9]. Furthermore, specific changes in the brain structure have been observed in depressed older adults. There is a significant reduction in the volume of various areas, including the caudate, putamen, anterior cingulate cortex, and notably the hippocampus. The latter appears to be a likely consequence of impaired neurogenic processes. Additionally, white matter hyperintensities, believed to result from small, silent cerebral infarctions causing demyelination and axonal degeneration, have been identified. Evidence suggests that the decreased integrity of white matter tracts, indicating dysfunctional brain networks, is associated with reduced executive function—a common feature in late-life depression. This decreased integrity is also linked to impairments in emotion regulation, attention, salience detection, and self-referential thinking [10, 11]. All the aforementioned peculiarities of geriatric depression have substantial implications for the onset of neurodegenerative diseases [12] and an even greater influence on the risk of suicidal attempts [3].

The prevailing theory underpinning the development of MDD is the “monoamine hypothesis”, which suggests that a reduction in monoamine neurotransmitters plays a central role in its pathogenesis [13]. Accordingly, the primary choice for antidepressant therapy includes selective serotonin/noradrenaline reuptake inhibitors. Nevertheless, one-third of older adults experience treatment resistance, resulting in an unfavorable prognosis characterized by compromised medical outcomes, heightened disability, and accelerated cognitive decline [14]. Individuals aged 65 years or older with MDD may also experience adverse events, including an elevated rate of falls [15], necessitating therapy withdrawal [16]. Additionally, it is essential not to underestimate the challenges stemming from polypharmacy [17], a prevalent condition in the older population.

Considering the reduced effectiveness and potential risks associated with traditional pharmacological therapy, evaluating innovative approaches has become imperative. This review will focus on the potential of microRNAs (miRNAs) that target Brain-Derived Neurotrophic Factor (BDNF) as innovative therapeutic candidates for addressing MDD in older adults. Special emphasis will be placed on the delivery methods, comparing the strengths and weaknesses of each suggested approach.

## BDNF and MDD: from pathophysiology to therapy follow-up

As a member of the neurotrophin family, BDNF is significantly involved in the functioning of both developing and adult nervous systems. While neurons are the main producers of this molecule, its synthesis receives contributions from glial cells and various peripheral sources, with platelets acting as the primary storage reservoir [18]. In contrast to other components implicated in synapse function, BDNF stands out not only as a modulator but also as a pivotal mediator of synaptic plasticity and communication [19]. Consequently, deficiencies in BDNF-related signaling exert a profound influence on the brain, contributing to the development and exacerbation of neurological and psychiatric disorders.

In MDD, a significant decrease in both central and peripheral BDNF has been documented, with potential implications for therapy response as well [20, 21]. In the limbic regions of depressed suicide patients, both BDNF mRNA and protein levels exhibit significant reductions, while studies on animal models have further demonstrated that chronic exposure to stressful stimuli triggers the downregulation of this neurotrophin in key areas, such as the hippocampus and nucleus accumbens [22]. Reduced levels of BDNF have been shown to affect neuronal structure, leading to a decrease in dendritic arbor complexity and a simultaneous increase in behavioral abnormalities [23]. Beyond the neurotrophin quantity, the interaction with Tropomyosin receptor kinase B (TrkB), the receptor through which BDNF engages in a ligand-specific manner, holds pivotal significance. Preclinical studies have demonstrated improvements in the depressive-like profile when the BDNF/TrkB signaling pathway is upregulated [24–26]. Interestingly, the use of subanesthetic doses of ketamine, employing a hormetic approach, has been receiving growing attention in recent years as a novel treatment for MDD [27]. Ketamine, by blocking N-methyl-D-aspartate receptors of glutamate, induces a molecular cascade that precisely promotes the release of BDNF and then, via the BDNF/TrkB/mTORa pathway, enhances neuronal plasticity, facilitated by neurotransmitter homeostasis, synapse recovery, and an increase in dendritic spines [28].

Apart from the well-documented changes in BDNF metabolism in the brain, several meta-analyses revealed a correlation between increased blood levels of BDNF and symptom improvement in individuals with MDD responding to pharmacological therapies, regardless of treatment modality [29, 30]. Even more clinically relevant, there is evidence regarding the potential to integrate blood BDNF levels with Hamilton Depression Rating Scale scores to predict treatment response during the early phases of therapy, prior to observable symptom changes [31]. Finally, in the context of late-onset MDD, it is crucial to emphasize that within a particularly vulnerable segment of the elderly population, specifically individuals affected by Mild Cognitive Impairment, blood BDNF has been identified as a correlative and prognostic factor for the decline in mood status [32].

## The role of miRNA deregulation in MDD: insights into neuroplasticity, inflammation, and BDNF

miRNAs are relatively small (19–25 nucleotides in length) non-coding single stranded RNAs. Typically, these molecules play a regulatory role by suppressing expression through interaction with the 3′ UTR of target messenger RNAs (mRNAs). However, their influence extends to diverse regions, including the 5′ UTR, coding sequence, and gene promoters. Remarkably, miRNAs challenge the conventional suppressive role; under specific conditions, they act not only as inhibitors but also as activators of gene expression [33]. A few miRNAs possess the capability to target hundreds of mRNAs, thereby regulating the expression of numerous genes [34, 35]. Approximately 70% of miRNAs have been found in the brain [36–38] and, although only a handful of them are expressed in a brain-enriched or brain-specific manner [39], they all play pivotal roles in various functions, including neurogenesis, regulation of axonal morphology, synaptic formation, astrocyte differentiation, as well as cellular apoptosis, proliferation, and migration [40]. It is therefore not surprising that their deregulation has been documented in numerous neurological and psychiatric diseases [41, 42], including MDD (Table 1).

**Table 1:** A summary of specific miRNAs whose dysregulation is recognized to play a role in major depressive disorder.

miRNA	MDD	Molecular target	Affected pathways	References
miR-185 miR-491-3p	Up regulated^a^	TrkB-T1	Neurogenesis, synaptogenesis, neuroplasticity	[90, 91]
miR-30e miR-132 miR-212	Up regulated^b^	BDNF	Neurogenesis, synaptogenesis, neuroplasticity	[92]
miR-323a-3p	Up regulated^a^	ERBB4	Synaptogenesis	[93]
miR-204-5p	Up regulated^a^	EPHB2, TrkB, ERBB3/4	Synaptogenesis/neuroplasticity	
miR-320b	Up regulated^a^	DLX5, ERBB4	Neurogenesis	
miR-331-3p	Up regulated^a^	ERBB2, NRP-2	Neurogenesis and neuroplasticity	
miR-134	Down regulated^b^	CREB	Neurogenesis/synaptic plasticity	[94]
miR-10a-5p miR-374b-5p	Down regulated^c^	not yet defined	Neuroplasticity/neurotrophy	[95]
miR-542-3p miR-181-3p miR-3690	Down regulated^b^ Down regulated^b^	PTGS2 NRF2, eIF4E, CXCL8	Neuroinflammation/neuroplasticity neuroinflammation	[96]
miR-146a miR-155	Down regulated^c^	TLR4	Inflammation	[97, 98]
miR-29a	Down regulated^b^	TLR8	Inflammation	[99]

^a^Post mortem human brain.

^b^Human blood.

^c^Peripheral blood mononuclear cells.

The exhaustive listing of all miRNAs identified to date with a role in the pathophysiology of MDD exceeds the scope of this review. Consequently, the presented table should be considered a partial selection aiming to be as representative as possible of the overall scenario. Nevertheless, a discernible pattern emerges, indicating that most of the miRNAs implicated in MDD onset and progression regulate proteins directly involved in neuroplasticity. Besides, a deficit in neuronal adaptation may detrimentally affect an individual’s capacity to cope with highly stressful events and consequently contributing to the development of depression [43]. Recent evidence also underscores the potential role of miR-218 in contributing to the dysregulation of the hypothalamic-pituitary-adrenal (HPA) axis, the pivotal neuroendocrine system governing the physiological response to stress, with significant consequences in MDD [44]. miR-218 may exacerbate the well-documented impairment of the HPA axis throughout the aging process [45], which not only directly influences the onset of the disease [46, 47] but also has adverse effects on hippocampal plasticity [48]. Nevertheless, inflammation also emerges as a significant target of miRNAs in MDD. Besides, many miRNAs listed in Table 1 have been implicated in the promotion of cellular senescence, such as miR-185 [49], or demonstrated to be either upregulated (miR-134) or downregulated (miR-146a and miR-155) in this context [50]. Senescent cells, via their senescence-associated secretory phenotype (SASP), constitute a significant source of pro-inflammatory mediators during aging and age-related diseases [51], including central nervous system (CNS) pathologies influenced by SASP factors released by non-neuronal brain cells [52, 53]. During aging, astrocytes increase the release of TNF-α, IL-1β, and IL-6, readily assuming the senescence phenotype after a stimulus [54]. Similarly, aged microglia overexpress proinflammatory cytokines and exhibit reduced responsiveness to anti-inflammatory molecules such as IL-10, thereby exacerbating cognitive impairment, sickness, and depressive-like behaviors [55]. In addition to the individual impact of the deregulation of neuroplasticity and neuroinflammation, emerging findings highlight the interplay between these two phenomena in the etiopathology of MDD. Pro-inflammatory cytokines reduce the bioavailability of specific neurotransmitters (e.g., serotonin, norepinephrine, and dopamine), leading to an imbalance between glutamatergic excitation and GABAergic inhibition and disrupt neurocircuitry implicated in the pathogenesis of depression (e.g., basal ganglia and anterior cingulate cortex regions) [56]. In the complex interaction between miRNA-induced neuroplasticity deficits and neuroinflammation, BDNF could be a key player.

The detrimental impact of reduced brain BDNF levels and deficit in its functional pathway on neuroplasticity is easily comprehensible [57, 58], based on the biological processes in which this neurotrophin is involved, spanning from neuronal survival and differentiation to synaptic functioning and cognitive performances [59]. The issue is further compounded by the excessive production of glucocorticoids resulting from HPA axis dysfunction, which significantly diminishes BDNF presence, as activated glucocorticoid and mineralocorticoid receptors repress the transcription activity of the BDNF promoter site [60]. The connection with neuroinflammation may appear more intricate. Nevertheless, recent research has unveiled a bidirectional modulation between BDNF and neuroinflammation [61]: chronic inflammation disrupts the long-term expression of BDNF and decreased BDNF levels, in turn, lead to abnormal microglia activation in response to stimuli, resulting in various effects, including damage to the blood–brain barrier (BBB). The vicious cycle among inflammatory molecules and BDNF appears to be exacerbated in the presence of the Val66Met genotype [62], a polymorphism of BDNF widely evaluated as a possible susceptibility factor for the development of MDD [63]. Interestingly, experimental stimulation of neuroinflammation through LPS administration has been demonstrated to induce synaptic protein loss via the BDNF/TrkB pathway, establishing a proven causal relationship with the onset of depressive-like behavior [64]. In Supplementary Table 1, we have added further information concerning the miRNAs whose changes impact BDNF synthesis and signaling in late-onset MDD.

A challenging interventional hypothesis posits that the modulation of BDNF holds the potential to address neuroplasticity and neuroinflammation, thereby mitigating the deficits that contribute to the initiation and exacerbation of late-onset MDD. Compellingly, the administration of exogenous BDNF into the brain has been demonstrated to effectively downregulate proinflammatory cytokines such as TNF-α, IL-1β, and IL-6, while upregulating anti-inflammatory cytokines like IL-10, thus improving clinical outcomes in rodent models of stroke and meningitis [65, 66]. Similar results could be achieved in MDD.

## Reasons for exploring miRNA as a potential treatment for MDD in the older adults

As previously speculated, the administration of exogenous BDNF may elicit positive effects on neuroplasticity and neuroinflammation in the treatment of MDD. Nevertheless, we posit that employing miRNAs as therapeutic agents offers a more advantageous approach. BDNF faces limitations in crossing the BBB effectively and has a short half-life of only a few minutes [67], necessitating local administration, a method that proves highly complex to implement in a clinical context. Therefore, miRNAs hold the potential for a safer and more effective strategy for both exerting their beneficial effects and facilitating CNS delivery.

As of 2022, a total of 17 RNA-based therapeutics, including mRNA, small interfering RNA (siRNA), antisense oligonucleotides (ASOs), and aptamers, have received approval from either the Food and Drug Administration or the European Medicines Agency. Additionally, there are approximately 222 RNA-based therapeutics currently in various stages of clinical trials [68], including numerous miRNA therapeutics (e.g., NCT01646489, NCT0120042 and NCT01872936 (Hepatitis C Virus), NCT04536688 (Autosomal Dominant Polycystic Kidney Disease), NCT03601052 (keloid patients)).

Utilizing miRNAs as tools offers advantages over other RNA-based approaches. Firstly, miRNAs are molecules physiologically present in human cells, facilitating their seamless integration into cellular machinery. Secondly, miRNAs have the capability to target multiple genes within the same pathway, resulting in a broader yet finely tuned response [69].

**Fig. 1:**
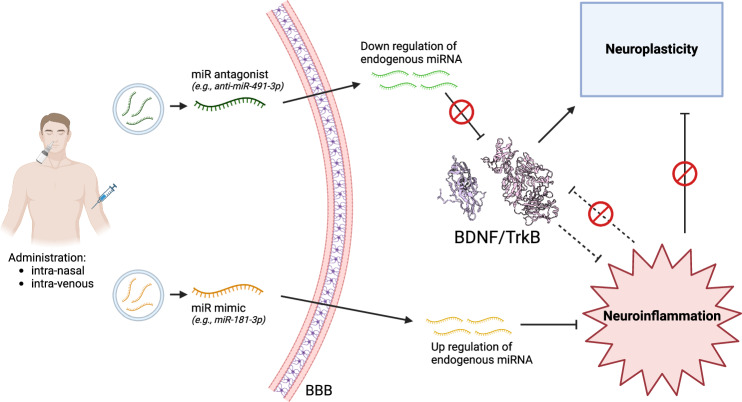
A proposal for the therapeutic use of miRNAs in treating major depressive disorder targeting BDNF. The scheme involves the simultaneous administration, either intravenously or intranasally, of synthetic miRNA molecules designed to target specific endogenous miRNAs that regulate BDNF and its interaction with TrkB (e.g., miR-491-3p), as well as miR-181-3p, which indirectly influences neurotrophin functioning. Both antagonistic and mimic miRNAs are employed. This approach holds the potential to reduce neuroinflammation and enhance neuroplasticity by up-regulating BDNF levels and its activity. BBB blood–brain barrier, BDNF brain-derived neurotrophic factor, TrkB tropomyosin receptor kinase B.

At present, two commonly utilized strategies leverage the therapeutic potential of miRNAs: replacement and inhibition. Replacement involves reinstating downregulated endogenous miRNAs by introducing synthetic miRNAs with identical sequences. Conversely, inhibition entails introducing antagonistic molecules capable of reducing the expression of endogenous miRNAs [70]. In Fig. 1, a schematic representation of a potential therapeutic approach is provided, which involves the replacement or inhibition of miRNAs that target BDNF, either directly or indirectly.

## Considerations for choosing miRNA vectors

A crucial concern lies in the choice of an appropriate delivery system for miRNAs. Viral vectors loaded with miRNAs have been extensively utilized, especially in cancer therapy. However, they present inherent challenges, such as cytotoxicity, carcinogenicity, and immunogenicity; on the contrary, non-viral delivery methods pose lower toxicity risks and are more compatible [71, 72].

Liposomes are lipid-based nanoparticles (NPs) that spontaneously assemble into spherical structures, comprising both single-layered and multilayered vesicles [73]. Notably, liposomes can encapsulate a wide range of payloads, both hydrophilic and hydrophobic, with considerable loading efficiency. In the case of hydrophilic compounds like miRNAs, this efficiency is additionally found to exhibit a positive correlation with vesicle size, typically falling within the range of 50 to 500 nm, while showing a negative correlation with the number of bilayers [74]. Despite their remarkable efficacy, liposome nanomedicine technology still exhibits certain limitations; nonetheless, many of these challenges can be addressed through strategic engineering of the liposome membrane. Indeed, one of the most intriguing features of these NPs is their high degree of customizability, surpassing conventional liposomes, including PEGylated and ligand-targeted liposomes [75]. To date, among all synthetic NPs, liposomes stand out as the safest and most versatile [76].

Exosomes are naturally occurring lipid extracellular vesicles, usually measuring between 30 to 200 nm in diameter [77]. They are generated during endosomal maturation and subsequently released into the extracellular space, carrying a diverse range of molecules, including DNA, RNA, proteins, and lipids [78]. Exosomes possess several characteristics that make them suitable as vehicles for miRNAs including circulation stability, biocompatibility, reduced immunogenicity, low toxicity, and the capability to traverse the BBB [79, 80]. However, despite these advantages, there are still certain shortcomings, which, also in this case, can be addressed through specific chemical engineering.

Polyethyleneimines (PEIs) are synthetic macromolecules with positive charges either in the polymer backbone or within their side chains. PEIs can effectively penetrate the CNS by bypassing the BBB [81] and their positive amine groups specifically bind to anionic RNA, thereby protecting it from degradation and facilitating cellular uptake [82]. These unique characteristics make PEIs the “gold standard” polymer for miRNA delivery [83]. However, the issue of toxicity, especially neurotoxicity, continues to pose a significant challenge in clinical applications [81], despite ongoing efforts to address it.

Finally, several inorganic materials have demonstrated their capacity for miRNA delivery, including chemically modified gold [84], iron oxide [85], and silica-based materials [86], among others. These compounds are typically highly stable and can be extensively engineered to enhance biocompatibility and enable precise targeting. Indeed, metal core NPs can be synthesized with such a high degree of control that they create uniform ultrasmall drug delivery vehicles (<100 nm) with exceptionally advantageous characteristics, although some concern arises about the long-term consequences, especially at high dosages.

**Fig. 2:**
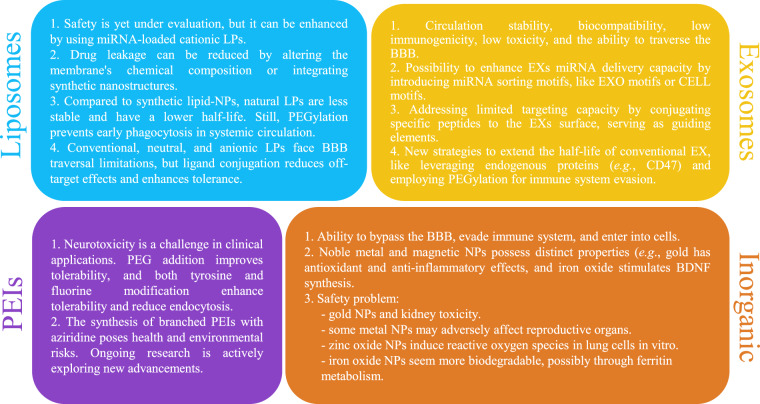
Schematic view of advantages and major drawbacks of artificial vehicles for miRNA delivery. Lipid-based nanoparticles (NPs), i.e., liposomes [100–107] and exosomes [79, 80,108–111], offer a high degree of safety, customization, and versatility in terms of encapsulating hydrophilic payloads, including miRNAs. However, they can be susceptible to drug leakage, exhibit relatively shorter half-lives, and face challenges in achieving specific targeting. On the other hand, Polyethyleneimines (PEIs) [81, 112–115] and inorganic materials (i.e., gold and iron oxide NPs) [116–121] provide stability and can be engineered for precise targeting, but potential long-term toxicity concerns and limited biodegradability warrant careful consideration. Collectively, inorganic NPs exhibit considerable potential in miRNA therapeutic delivery. Nevertheless, we assert that their limitations surpass their potential advantages, whereas lipid-based carriers show promise owing to their safety profile and customizability, along with the possibility of implementing modifications to mitigate their drawbacks. CNS central nervous system, BBB blood–brain barrier, PEG polyethylene glycol.

Figure 2 provides a more detailed overview of the strengths and weaknesses within each category. While not claiming to be exhaustive, it can serve as a starting point for making sound choices.

## Conclusions

In older adults, MDD significantly affects daily life, particularly by severely impairing neurocognitive functions. This leads to elevated rates of disability and mortality when compared to age-matched non-depressed individuals [87]. Mood disorders in the elderly population also impose a significant economic and logistical burden on healthcare systems [88]. Exploring new therapeutic approaches, either as a supplement to standard guidelines or as alternatives for non-responsive patients, could pave the way for improved management of geriatric MDD patients.

In recent years, the increasing focus on BDNF alterations and their involvement in the onset and progression of MDD has led to growing interest, giving rise to the “neurotrophin hypothesis of depression“ [89]. Targeting BDNF expression through tailored miRNAs stands out as a potentially innovative approach in pathology oversight. Two essential steps are required: firstly, conducting a comprehensive and as exhaustive as possible analysis of the miRNA molecules involved in BDNF pathways and their relationship to MDD, and secondly, identifying the optimal delivery vehicle. Concerning the first aspect, the simultaneous utilization of multiple miRNAs capable of directly influencing the neurotrophin while also activating other closely associated pathways, such as neuroinflammation, represents, in our opinion, the ideal approach. As for the second topic, while the perfect vehicle has not been developed yet, considering the trade-offs, liposomes appear to be the most appropriate choice based on their current properties.

One last topic that deserves specific consideration is the current technical incapacity to exclusively target the brain areas involved in the onset and progression of MDD. Given the pleiotropic functions of BDNF, altering its synthesis and signaling in areas without impairments could pose a significant side effect. Investigating potential molecular ‘footprints’ of neurons and glial cells of depressed patients in the most affected areas, such as the hippocampus, should be the primary research objective. This would allow equipping vectors with high-affinity ligands for these molecules. Furthermore, even though the majority of BDNF is synthesized within the CNS, there are also peripheral sources [18]. To minimize systemic alterations, the inhalation route of administration is preferable to the intravenous one.

## Supplementary information

Supplementary Table 1
