# Supplementary material for: Late-onset major depressive disorder: exploring the therapeutic potential of enhancing cerebral brain-derived neurotrophic factor expression through targeted microRNA delivery
Source: Transl Psychiatry. 2024 Sep 3;14:352. doi: 10.1038/s41398-024-02935-7 (PMC11371930; doi:10.1038/s41398-024-02935-7)
Supplement: Supplementary file 1 — Supplementary Table 1 [file 41398_2024_2935_MOESM1_ESM.docx]

| **Dysregulated TrkB/BDNF-targeting miRNAs in MDD** | **Species** | **Affected brain regions** | **Observed abnormalities** |
| --- | --- | --- | --- |
| *miR-185* **[1]** | Human | Anterior Prefrontal Cortex | Capillary dysfunction **[2]**. |
| *miR-30e* **[3, 4]** | Human, mouse | Dorso-lateral Prefrontal Cortex (dlPFC)  Dentate gyrus | Reduced volume and blood flow support **[5]**.  Reduced volume **[6,7]**, reduced BDNF expression **[8]**. |
| *miR-132* **[9, 10]** | Rat | dlPFC  Hippocampus | Reduced volume and blood flow support **[5]**.  Reduced volume of whole left hemisphere hippocampus **[11-13]**. |
| *miR-212* **[14]** | Mouse | Hippocampus | Reduced volume of whole left hemisphere hippocampus **[11-13]**. |
| *miR-491-3p* **[1]** | Human | Anterior Prefrontal Cortex | ​Capillary dysfunction **[2]**. |
| *miR-204-5p* **[15, 16, 17]** | Human, mouse, rat | Anterior Cingulate Cortex (ACC)  Dentate gyrus  Lateral habenula | Impaired functional connectivity between ACC and dlPFC and amygdala **[18]**.  Neuronal atrophy and reduced volume **[19]**.  Reduced cortical thickness **[20]**.  Reduced volume **[6, 7]**, reduced BDNF expression **[8]**.  Increased blood flow **[21]**. |

**Supplementary Table 1.** **Examples of miRNAs deregulated in late-onset Major Depressive Disorder (MDD) with a documented role in BDNF synthesis and signaling: details regarding the affected brain areas and structural/functional consequences.**

**References**

1 Maussion G, Yang J, Yerko V, Barker P, Mechawar N, Ernst C et al. Regulation of a truncated form of tropomyosin-related kinase B (TrkB) by Hsa-miR-185* in frontal cortex of suicide completers. PLoS One 2012;7: e39301.

2 Dalby RB, Eskildsen SF, Videbech P, Rosenberg R, Østergaard L. Cerebral hemodynamics and capillary dysfunction in late-onset major depressive disorder. Psychiatry Res Neuroimaging 2021; 317: 111383.

3 Gorinski N, Bijata M, Prasad S, Wirth A, Abdel Galil D, Zeug A et al. Attenuated palmitoylation of serotonin receptor 5-HT1A affects receptor function and contributes to depression-like behaviors. Nat Commun 2019; 10: 3924.

4 Khandelwal N, Dey SK, Chakravarty S, Kumar A. miR-30 Family miRNAs Mediate the Effect of Chronic Social Defeat Stress on Hippocampal Neurogenesis in Mouse Depression Model. Front Mol Neurosci 2019; 12: 188.

5 Galynker II, Cai J, Ongseng F, Finestone H, Dutta E, Serseni D. Hypofrontality and negative symptoms in major depressive disorder. J Nucl Med 1998; 39: 608–612.

6 Travis S, Coupland NJ, Silversone PH, Huang Y, Fujiwara E, Carter R et al. Dentate gyrus volume and memory performance in major depressive disorder. J Affect Disord 2015; 172: 159–164.

7 Boldrini M, Santiago AN, Hen R, Dwork AJ, Rosoklija GB, Tamir H et al. Hippocampal granule neuron number and dentate gyrus volume in antidepressant-treated and untreated major depression. Neuropsychopharmacology 2013; 38: 1068–1077.

8 Li Y, Ji Y, Jiang H, Liu D, Zhang Q, Fan S et al. Effects of unpredictable chronic stress on behavior and brain-derived neurotrophic factor expression in CA3 subfield and dentate gyrus of the hippocampus in different aged rats. Chin Med J (Engl) 2009; 122: 1564–1569.

9 Su M, Hong J, Zhao Y, Liu S, Xue X. MeCP2 controls hippocampal brain-derived neurotrophic factor expression via homeostatic interactions with microRNA‑132 in rats with depression. Mol Med Rep 2015; 12: 5399–5406.

10 Tong L, Li M-D, Nie P-Y, Chen Y, Chen Y-L, Ji L-L. miR-132 downregulation alleviates behavioral impairment of rats exposed to single prolonged stress, reduces the level of apoptosis in PFC, and upregulates the expression of MeCP2 and BDNF. Neurobiol Stress 2021; 14: 100311.

11 Wu C, Jia L, Mu Q, Fang Z, Hamoudi HJAS, Huang M et al. Altered hippocampal subfield volumes in major depressive disorder with and without anhedonia. BMC Psychiatry 2023; 23: 540.

12 Mervaala E, Föhr J, Könönen M, Valkonen-Korhonen M, Vainio P, Partanen K et al. Quantitative MRI of the hippocampus and amygdala in severe depression. Psychol Med 2000; 30: 117–125.

13 Travis SG, Coupland NJ, Hegadoren K, Silverstone PH, Huang Y, Carter R et al. Effects of cortisol on hippocampal subfields volumes and memory performance in healthy control subjects and patients with major depressive disorder. J Affect Disord 2016; 201: 34–41.

14 Si L, Wang Y, Liu M, Yang L, Zhang L. Expression and role of microRNA-212/nuclear factor I-A in depressive mice. Bioengineered 2021; 12: 11520–11532.

15 Fiori LM, Kos A, Lin R, Théroux J-F, Lopez JP, Kühne C et al. miR-323a regulates ERBB4 and is involved in depression. Mol Psychiatry 2021; 26: 4191–4204.

16 Guan W, Wu X-Y, Jin X, Sheng X-M, Fan Y. miR-204-5p Plays a Critical Role in the Pathogenesis of Depression and Anti-depression Action of Venlafaxine in the Hippocampus of Mice. Curr Med Chem 2023.

17 Lan T, Li Y, Fan C, Wang L, Wang W, Chen S et al. MicroRNA-204-5p reduction in rat hippocampus contributes to stress-induced pathology via targeting RGS12 signaling pathway. J Neuroinflammation 2021; 18: 243.

18 Zhang Y, Shao J, Wang X, Chen Z, Liu H, Pei C et al. Functional impairment-based segmentation of anterior cingulate cortex in depression and its relationship with treatment effects. Hum Brain Mapp 2021; 42: 4035–4047.

19 Koolschijn PCMP, van Haren NEM, Lensvelt-Mulders GJLM, Hulshoff Pol HE, Kahn RS. Brain volume abnormalities in major depressive disorder: a meta-analysis of magnetic resonance imaging studies. Hum Brain Mapp 2009; 30: 3719–3735.

20 Kang Y, Shin D, Kim A, You S-H, Kim B, Han K-M et al. The effect of inflammation markers on cortical thinning in major depressive disorder: A possible mediator of depression and cortical changes. J Affect Disord 2024; 348: 229–237.

21 Morris JS, Smith KA, Cowen PJ, Friston KJ, Dolan RJ. Covariation of activity in habenula and dorsal raphé nuclei following tryptophan depletion. Neuroimage 1999; 10: 163–172.
